# Supplementary material for: Temporal and spatial features of the palace building space of Qinghai’s Kumbum Monastery and its evolution
Source: PLoS One. 2022 Oct 20;17(10):e0262155. doi: 10.1371/journal.pone.0262155 (PMC9584376; doi:10.1371/journal.pone.0262155)
Supplement: Attachment — Submitted filename: Response to Reviewers.docx [file pone.0262155.s003.docx]

**Dear Editors and Reviewers,**

Thank you for your letter and for the reviewers’ comments concerning our manuscript entitled “Temporal and spatial features of the palace building space of Qinghai’s Kumbum Monastery and its evolution mechanism” (PONE-D-21-25675).

Your comments and those of the reviewers were highly insightful and enabled us to greatly improve the quality of our manuscript. In the following pages are our point-by-point replies to each of the comments of the reviewers.

Revisions in the text are shown using yellow highlight for additions. We hope that the revisions in the manuscript and our accompanying responses will be sufficient to make our manuscript suitable for publication in Advances in PLOS ONE.

We shall look forward to hearing from you at your earliest convenience.

Yours sincerely,

Jing Zhang

Department of Environmental Design, School of Art and Media, Xi'an Technological University

Xi'an, Shaanxi, China

Email: ph_zhangjing@163.com

**Replies to the Journal Requirements:**

***Comments1:***

Please ensure that your manuscript meets PLOS ONE's style requirements, including those for file naming. The PLOS ONE style templates can be found at [https://journals.plos.org/plosone/s/file?id=wjVg/PLOSOne_formatting_sample_main_body.pdf](https://journals.plos.org/plosone/s/file?id=wjVg/PLOSOne_formatting_sample_main_body.pdf) and
[https://journals.plos.org/plosone/s/file?id=ba62/PLOSOne_formatting_sample_title_authors_affiliations.pdf](https://journals.plos.org/plosone/s/file?id=ba62/PLOSOne_formatting_sample_title_authors_affiliations.pdf)

***Reply:***

Thank you for your valuable comments on our paper. According to the PLOS ONE templates, we revised our manuscript to meet PLOS ONE ’s style requirements.

***Comments2:***

In your manuscript, please provide additional information regarding the specimens used in your study. Ensure that you have reported specimen numbers and complete repository information, including museum name and geographic location.

If permits were required, please ensure that you have provided details for all permits that were obtained, including the full name of the issuing authority, and add the following statement: 'All necessary permits were obtained for the described study, which complied with all relevant regulations.'

If no permits were required, please include the following statement: 'No permits were required for the described study, which complied with all relevant regulations.'

***Reply:***

Thank you for your valuable comments on our paper. We add the statement with 'No permits were required for the described study, which complied with all relevant regulations.'

due to no permits were required.

***Comments3:***

We note that Figures 1 and 3 in your submission contain [map/satellite] images which may be copyrighted…We require you to either (1) present written permission from the copyright holder to publish these figures specifically under the CC BY 4.0 license, or (2) remove the figures from your submission…. If you are unable to obtain permission from the original copyright holder to publish these figures under the CC BY 4.0 license or if the copyright holder’s requirements are incompatible with the CC BY 4.0 license, please either i) remove the figure or ii) supply a replacement figure that complies with the CC BY 4.0 license.

***Reply:***

Thank you for your valuable comments on our paper. First, Figure 1 has been removed in the revised manuscript.

Second, Figure 3, we supplied a replacement figure (Fig 2) which is the schematic diagram just showing the distribution of buildings, and all the information in this replacement figure are based on our site investigation (see attachment ).

***Comments4:***

We note that Picture 1 in your submission contain copyrighted images…We require you to either (1) present written permission from the copyright holder to publish these figures specifically under the CC BY 4.0 license, or (2) remove the figures from your submission:

***Reply:***

Thank you for your valuable comments on our paper. Pic 1 has been removed in the revised manuscript.

**Replies to the comments of Reviewer #1**

***Comment 1:***

Structure. Literature Review had better be separated from the Introduction and make a separate section. Study Objects and Methodology should be placed in a separate section of “Methodology”. Background Overview is the content about the study area. It should also be in the “Methodology” section. The above is only the suggestion, please adjust the structure according to the actual situation.

Table. Please pay attention to the format. For example, 3-line table is usually required in published articles.

The clarity of all figures needs to be improved, and some formats are wrong as shown in Fig.2. Please provide the original figures in the attachment.

***Reply 1:***

Thank you for your valuable comments on our paper.

Literature Review has been separated from the Introduction and make a separate section. Moreover, Study Objects and Methodology, and Background Overview have been placed in a separate section of “Methodology”.

All the Table have been revised in the standard format according to PLOS ONE Table Guidelines. Among these Tables, Table 2-3, and 5-7 in preliminary submission have been transfer into Figure 5-7, 9 and 12 because of containing graphics (see attachment).

Figure 2 has been replaced (Fig 1 now) (see attachment).

***Comment 2:***

In "4.1 Spatial and temporal Evolution and features of the Palace Buildings", I hope to see the evolution of the features of palace Buildings on a map/diagram, but you only gave the pie chart. In view of “Spatial and temporal evolution”, considering adding meaningful maps or modifying this subtitle.

Also, the language needs to be polished by at least one native speakers.

***Reply 2:***

Thank you for your valuable comments on our paper. In "Spatial and temporal Evolution and features of the Palace Buildings", we add Fig 14 to show the distribution of the four types of palace spaces in buildings examples (see attachment). And the language has been polished by a native speaker.

**Replies to the comments of Reviewer #2**

***Comment 1:***

The structure of the article is chaotic and the writing pattern is not scientific enough. The current structure is difficult to read and cannot form a smooth logical system. It is hoped that the manuscript can be reorganized in the order of Introduction - Methods - Results and discussion – Conclusion.

***Reply 2:***

Thank you for your valuable comments on our paper. According to your and reviewer 1’s precious suggestions, the structure of the article has been reorganized in the order of Introduction – Literature review -Materials and methods –Results and discussion – Conclusion.

***Comment 2:***

The introduction of research methods is relatively thin. The rationality of the method is the guarantee of the scientificity of the article. The author should introduce the use methods of various materials in detail, while the current introduction is too general.

This paper has almost no discussion on typology. I don't know how the author uses the typological method to analyze. It is speculated from the article that the author should want to introduce the morphological evolution of buildings in Kumbum Monastery with the method of process typology, but it is lack of accurate definition.

In addition, how is the importance of numerical analysis reflected in the research？ I think the author should strengthen the interactive analysis of this data and the evolution mechanism of building types, not just a simple list.

***Reply 2:***

According to your suggestion, we revised our article as follows:

1.We add Fig 3. (Research method framework) to show the research methods of various materials in detail (See attachment).

2. We explain the classification criteria and basis based on typology in “Spatial composition types and features of the palace buildings” (see line 117-124, whose supporting information can be seen in line 98-104) and in” Spatial composition types and features of the palace buildings” (see line 275-279). Also, we gave the definition of 4 types of palace spaces (type1,see line 283-284; type2, see line 315-317; type3, see line 343-345; type4, see line 378-379).

3. Fig 14 has been supplied to show the distribution of the four types of palace spaces in buildings examples. And Fig 14 has been added to strengthen the evolution mechanism of building types in historical context (see attachment).

***Comment 3:***

I think section 2, 3 and 4 of the article can be integrated for in-depth discussion without parallel listing. At present, the introduction of historical narration and architectural features are independent and lack of organic connection.

***Reply 3:***

Thank you for your valuable comments on our paper. “2.1 Development Background of Tibetan Buddhism in Qinghai” in preliminary submission has been removed, and been separated and connected with the certain parts in “Temporal and spatial evolution” (see line 458-464 and line 481-496) to integrated for in-depth discussion.

***Comment 4:***

The research on historical buildings focuses on the protection and utilization, but the article just lacks too much content on this point. The theoretical and practical significance of the research should not be ignored. What guiding value does the research results have for the protection of buildings in Kumbum Monastery? What measures should be taken to continue the tradition of Tibetan Buddhist architecture in the future? These should be described in detail in the discussion section.

***Reply 4:***

Thanks for your valuable comments. Though the protection and utilization of Tibetan Buddhist monastery is crucial , its specific measurements in details are the further aim of its research, and it is not the direct research purpose of this article yet. Meanwhile, your suggestion is quite meaningful to us. A brief statement of guiding value, and protection and conversation principle of Kumbum Monastery and other Tibetan Buddhist monasteries in Qinghai in the future has been supplied in the discussion section ( see line 519-526).

***Comment 5:***

The abstract should be revised according to the modification of the article, and at least include a brief description of the building type.

***Reply5:***

A brief description of the four building types have been explained in the abstract (see line 26-28).

***Comment 6:***

The main analysis and thinking can be put in the "Results and discussion", and the conclusion only needs to condense the main points of the article.

***Reply 6:***

All the main analysis sections have been organized in the "Results and discussion", as well as replaced the first part of “Conclusion and discussion” in original manuscript (see line 505-518).

***Comment 7:***

It is recommended that the influence mechanism can be omitted, focusing on the changes in the structure of the temple, and not discussing the influencing factors. Because there are many influencing factors, it is beyond the control of the author.

***Reply 7:***

Thanks for your valuable suggestions. We turned focus from evolution mechanism to evolution process, and changed the corresponding titles (see line4, 405,451) and the content (see line30,41,71,152,159,407).

***Comment 8:***

A brief discussion can be done through literature review.

***Reply 8:***

A brief discussion has been supplied through literature review (see line 98-104).

***Comment 9:***

here should be an explanation for terms such as Central Plains, not the Central Plains, but the representative name of the Chinese culture?

In addition, it is recommended to invite experts in geopolitics and international relations to review the language of the article.

***Reply 9:***

“Central Plains” has been modified and explained according to the explanation from “Ci hai”（The Chinese dictionary）and experts in geopolitics (Zhang,2007) (see line 473-476), as well as the “Reference” (see line 604-605).

**Replies to the comments of Reviewer #7**

***Comment 1:***

Most of the analyzed slopes are at the margin of instability i.e., the FS is round 1.

***Reply 1:***

Thank you for your valuable comments on our paper.

The evaluation method based on FE analysis proposed in this paper could consider the progressive failure characteristics of slopes. Therefore, in order to make the slope from stable to local instability and finally reaches a state of complete failure, the FoS of some schemes is round 1.0 (local instability) when designing the calculation schemes.

***Comment 2:***

It is interesting to examine some examples where the FS is high than 2. And accordingly the error is calculated and compared between the two methods. If the error is high then it is important to know.

It is observed that when the FS is higher than 1.5 to 2 the error is less than 10%.

***Reply 2:***

In this study, when $FoS>1.5$, the slope is basically in a stable state and the calculation error is within 10%. Since the state of sliding surface elements is completely fixed for the stable slope, the strength value brought into the FoS equation is peak strength, and the error is generally small.

***Comment 3:***

For practical purpose when the FS is around one or less, it is not that important to estimate the error.

***Reply 3:***

For an actual slope, it is obvious that the slope has been unstable when $FoS<1.0$, and the values of the FoS and error are not important. However, the purpose of this study is to explore the calculated error between two methods, the stress distribution of sliding surfaces and the progressive failure characteristics when slopes are in different states (the whole process), so some calculation cases with $FoS<1.0$ are also added.
