# Supplementary material for: Temporal and spatial features of the palace building space of Qinghai’s Kumbum Monastery and its evolution
Source: PLoS One. 2022 Oct 20;17(10):e0262155. doi: 10.1371/journal.pone.0262155 (PMC9584376; doi:10.1371/journal.pone.0262155)
Supplement: Attachment — Submitted filename: Response to Reviewers.docx [file pone.0262155.s004.docx]

**Dear Editors and Reviewers,**

Thank you for your letter and for the reviewers’ comments concerning our manuscript entitled “Temporal and spatial features of the palace building space of Qinghai’s Kumbum Monastery and its evolution mechanism” (PONE-D-21-25675).

First of all, we sincerely apologized. Due to the negligence of the working team, the content of other manuscripts was mixed at the end of the previous replies to the comments of Reviewer 2. In this regard, we have deeply criticized and revised, and strengthened the frequency of document proofreading to guarantee that this situation will never happen again . In any case, we must deeply reflect on the last mistake and sincerely apologize to Reviewer 2.

Your comments and those of the reviewers were highly insightful and enabled us to greatly improve the quality of our manuscript. In the following pages are our point-by-point replies to each of the comments of the reviewers.

Revisions in the text are shown using yellow highlight for additions. We hope that the revisions in the manuscript and our accompanying responses will be sufficient to make our manuscript suitable for publication in Advances in PLOS ONE.

We shall look forward to hearing from you at your earliest convenience.

Yours sincerely,

Jing Zhang

Department of Environmental Design, School of Art and Media, Xi'an Technological University

Xi'an, Shaanxi, China

Email: ph_zhangjing@163.com

**Replies to the comments of Reviewer #1**

***Comment ：***

All comments have been addressed.It is recommended for publication.

***Reply :***

Thanks for your valuable comments on our paper.

**Replies to the comments of Reviewer #2**

**First of all, we sincerely apologize. Due to the negligence of our working team, the content of other manuscripts was mixed at the end of the previous replies to the comments of Reviewer 2. In this regard, we have deeply criticized and revised, and strengthened the frequency of document proofreading to guarantee that this situation will never happen again. In any case, we must deeply reflect on the last mistake and sincerely apologize to Reviewer 2.**

***Comment 1:***

The structure of the article is chaotic and the writing pattern is not scientific enough. The current structure is difficult to read and cannot form a smooth logical system. It is hoped that the manuscript can be reorganized in the order of Introduction - Methods - Results and discussion – Conclusion.

***Reply 1:***

Thank you for your valuable comments on our paper. According to your and reviewer 1’s precious suggestions, the structure of the article has been reorganized in the order of Introduction – Literature review -Materials and methods –Results and discussion – Conclusion.

***Comment 2:***

The introduction of research methods is relatively thin. The rationality of the method is the guarantee of the scientificity of the article. The author should introduce the use methods of various materials in detail, while the current introduction is too general.

***reply 2:***

According to your suggestion, we revised the introduction, and introduced the use methods to guarantee of the scientificity of the article (see line 69-75). Also, we revised Fig 2. (Research method framework) to show the research methods of various materials in detail (See attachment).

***Comment 3:***

This paper has almost no discussion on typology. I don't know how the author uses the typological method to analyze. It is speculated from the article that the author should want to introduce the morphological evolution of buildings in Kumbum Monastery with the method of process typology, but it is lack of accurate definition.

***Reply 3:***

Thanks for your valuable comments on our paper. We have clairfied the research method using typology (see line 69-75), and have explained the classification criteria and basis based on typology in “Spatial composition types and features of the palace buildings” (see line 122-129, whose supporting information can be seen in line 103-109; and see line 141-143) and in” Spatial composition types and features of the palace buildings” (see line 281-285). Also, we gave the definition of 4 types of palace spaces (type1, see line 289-290; type2, see line 321-323; type3, see line 350-352; type4, see line 385-387).

***Comment 4:***

In addition, how is the importance of numerical analysis reflected in the research？ I think the author should strengthen the interactive analysis of this data and the evolution mechanism of building types, not just a simple list.

***Reply 4:***

Thanks for your valuable suggestion, we have analysed the number of each type cases, and the comparison of the number of different types of buildings, as well as the width-to-depth ratio of the main hall of 4 types (see line 443-448).Then we have added the analysis and the corresponding figure (Fig 15) to explore the evolution of 4 types of palace buildings through the interactive numerical analysis and historical context ( see attachment).

Furthermore, we have added the analysis and the corresponding figure (Fig 14) to discuss the distribution of 4 types of palace buildings to strengthen the interactive analysis of research objects and the overall space of Kumbum Monastery (see line 434-436, see attachment) .

***Comment 5:***

I think section 2, 3 and 4 of the article can be integrated for in-depth discussion without parallel listing. At present, the introduction of historical narration and architectural features are independent and lack of organic connection.

***Reply 5:***

Thank you for your valuable comments on our paper. “2.1 Development Background of Tibetan Buddhism in Qinghai” in preliminary submission has been removed, and been separated and connected with the certain parts in “Temporal and spatial evolution” (see line 479-485 and line 502-517) to integrated for in-depth discussion. Besides, we have added Fig 15 to show the architectural evolution with the historical context (see attachment).

***Comment 6:***

The research on historical buildings focuses on the protection and utilization, but the article just lacks too much content on this point. The theoretical and practical significance of the research should not be ignored. What guiding value does the research results have for the protection of buildings in Kumbum Monastery? What measures should be taken to continue the tradition of Tibetan Buddhist architecture in the future? These should be described in detail in the discussion section.

***Reply 6:***

Thanks for your valuable comments. According to the research result ,we have discussed the guiding value of the protection of buildings in Kumbum Monastery. We also explained the measures. And the protection and conversation principle of Kumbum Monastery and other Tibetan Buddhist monasteries in Qinghai in the future has been supplied in the discussion section (see line 541-552, and 560-562).

***Comment 7:***

The abstract should be revised according to the modification of the article, and at least include a brief description of the building type.

***Reply 7:***

A brief description of the four building types have been explained in the abstract (see line 26-28).

***Comment 8:***

The main analysis and thinking can be put in the "Results and discussion", and the conclusion only needs to condense the main points of the article.

***Reply 8:***

All the main analysis sections have been organized in the "Results and discussion", as well as replaced the first part of “Conclusion and discussion” in original manuscript (see line 526-539). And the discuss part has been separated (see line 541-552), as well as the Conclusions (see line 553-562).

***Comment 9:***

It is recommended that the influence mechanism can be omitted, focusing on the changes in the structure of the temple, and not discussing the influencing factors. Because there are many influencing factors, it is beyond the control of the author.

***Reply 9:***

Thanks for your valuable suggestions. We turned focus from evolution mechanism to evolution process, and changed the corresponding titles (see line4, 413,468) and the content (see line30,41,159,165,472).

***Comment 10:***

A brief discussion can be done through literature review.

***Reply 10:***

A brief discussion has been supplied through literature review (see line 103-109).

***Comment 11:***

There should be an explanation for terms such as Central Plains, not the Central Plains, but the representative name of the Chinese culture?

In addition, it is recommended to invite experts in geopolitics and international relations to review the language of the article.

***Reply 11:***

“Central Plains” has been modified and explained according to the explanation from “Ci hai”（The Chinese dictionary）and experts in geopolitics (Zhang, 2007) (see line 494-498), as well as the “Reference” (see line 632-633).
