# Supplementary material for: Temporal and spatial features of the palace building space of Qinghai’s Kumbum Monastery and its evolution
Source: PLoS One. 2022 Oct 20;17(10):e0262155. doi: 10.1371/journal.pone.0262155 (PMC9584376; doi:10.1371/journal.pone.0262155)
Supplement: Attachment — Submitted filename: Response to Reviewers.docx [file pone.0262155.s005.docx]

**Dear Editors and Reviewers,**

Thank you for your letter and for the reviewers’ comments concerning our manuscript entitled “Temporal and spatial features of the palace building space of Qinghai’s Kumbum Monastery and its evolution mechanism” (PONE-D-21-25675).

Your comments and those of the reviewers were highly insightful and enabled us to greatly improve the quality of our manuscript. In the following pages are our point-by-point replies to each of the comments of the reviewers.

Revisions in the text are shown using yellow highlight for additions. We hope that the revisions in the manuscript and our accompanying responses will be sufficient to make our manuscript suitable for publication in Advances in PLOS ONE.

We shall look forward to hearing from you at your earliest convenience.

Yours sincerely,

Jing Zhang

Department of Environmental Design, School of Art and Media, Xi'an Technological University

Xi'an, Shaanxi, China

Email: ph_zhangjing@163.com

**Replies to the comments of Reviewer #1**

***Comment ：***

All comments have been addressed.It is recommended for publication.

***Reply :***

Thanks for your valuable comments on our paper.

**Replies to the comments of Reviewer #2**

***Comment 1:***

The author has deleted some redundant contents and the structure of the article is clearer, but there are still some problems that will affect reading and understanding:

Line83-119, "Literature review" should be combined with the "Introduction". Through literature review, discuss the shortcomings of previous research, and lead to the problems to be solved in this paper. Figure 2 should be the content of "Materials and methods". I don't know why it appears in the first paragraph.

***Reply 1:***

Thank you for your valuable suggestions. We have combined “literature review” with the “Introduction” (see line 81-120). Among them, the discussion of shortcomings of previous research can be seen from line 100 to 106, and the relevant practical problems can be seen in line 106-112. Then the problem to be solved in this paper can be seen in line 113-120. Also, the former Figure 2 has been adjusted to the part of "Materials and methods",and renamed Figure 3 (see line 151 ,153and 154, and attachment Fig 3).

***Comment 2:***

Line167-274, these contents should not be placed in the "Materials and methods", but should appear in the section "Discussion".

***reply 2:***

According to your suggestion, we revised and replaced this part to the “Discussion” (see line 173-280).

***Comment 3:***

Line540-551, there are "Results and Discussions" before, and there is another paragraph of discussion here, which is very confusing. This part should be retitled and integrated into the "Results and Discussions".

***Reply 3:***

Thanks for your valuable comments on our paper. To prevent the confusion,we have removed the former level-3 heading“discussion” ,and integrated the content at the end of the "Results and Discussions".(see line 544-551).
