# Supplementary material for: Temporal and spatial features of the palace building space of Qinghai’s Kumbum Monastery and its evolution
Source: PLoS One. 2022 Oct 20;17(10):e0262155. doi: 10.1371/journal.pone.0262155 (PMC9584376; doi:10.1371/journal.pone.0262155)
Supplement: Attachment — Submitted filename: Response to Reviewers.docx [file pone.0262155.s006.docx]

**Dear Editors and Reviewers,**

Thank you for your letter and for the reviewers’ comments concerning our manuscript entitled “Temporal and spatial features of the palace building space of Qinghai’s Kumbum Monastery and its evolution mechanism” (PONE-D-21-25675).

Your comments and those of the reviewers were highly insightful and enabled us to greatly improve the quality of our manuscript. In the following pages are our point-by-point replies to each of the comments of the reviewers.

Revisions in the text are shown using yellow highlight for additions. We hope that the revisions in the manuscript and our accompanying responses will be sufficient to make our manuscript suitable for publication in Advances in PLOS ONE.

We shall look forward to hearing from you at your earliest convenience.

Yours sincerely,

Jing Zhang

Department of Environmental Design, School of Art and Media, Xi'an Technological University

Xi'an, Shaanxi, China

Email: ph_zhangjing@163.com

**Replies to the comments of Reviewer #1**

***Comment ：***

All comments have been addressed.It is recommended for publication.

***Reply :***

Thanks for your valuable comments on our paper.

**Replies to the comments of Reviewer #2**

***Comment 1:***

The authors have adequately addressed most of the comments raised in a previous round of review，but the references are not enough, some latest outcomes should be referred, for example: 1) The Effect of Climate Change on the Fall Foliage Vacation in China. Tourism Management. (On line doi：10.1016/j.tourman.2013.02.020); 2) The influence of high-speed rail on ice–snow tourism in northeastern China. Tourism Management（2020）, doi:10.1016/j.tourman.2019.104070; 3) Effects of rural revitalization on rural tourism. Journal of Hospitality and Tourism Management (2021), https://doi.org/10.1016/j.jhtm.2021.02.008.

***Reply 1:***

Thanks for suggestion.We have referred the latest outcomes in our paper.

1) The Effect of Climate Change on the Fall Foliage Vacation in China. Tourism Management.(see line94, and 592-594)
2) The influence of high-speed rail on ice–snow tourism in northeastern China.(see line110, and 620-622)
3) Effects of rural revitalization on rural tourism. (see line110-111, and 623-625)

***Comment 2:***

While revising your submission, please upload your figure files to the Preflight Analysis and Conversion Engine (PACE) digital diagnostic tool, https://pacev2.apexcovantage.com/. PACE helps ensure that figures meet PLOS requirements. To use PACE, you must first register as a user. Registration is free. Then, login and navigate to the UPLOAD tab, where you will find detailed instructions on how to use the tool. If you encounter any issues or have any questions when using PACE, please email PLOS at figures@plos.org. Please note that Supporting Information files do not need this step.

***Reply 2***

Thanks for suggestion.We have upload all figure files to the Preflight Analysis and Conversion Engine (PACE) digital diagnostic tool to ensure that figures meet PLOS requirements(see attachments).
